# 4192 Decreasing Inappropriate STAT Image Ordering

**DOI:** 10.1017/cts.2020.173

**Published:** 2020-07-29

**Authors:** Michael Cui, Jonathan Chung, Pritesh Patel, Ingrid Reiser

**Affiliations:** 1 University of Chicago

## Abstract

OBJECTIVES/GOALS: Currently physicians are able to order CT Chest/Abd/Pelvis images as STAT or Routine. STAT images denote an emergency and are done immediately. We aim to determine the percentage of CT images that are inappropriately ordered as STAT, determine physician image ordering habits, and develop targeted interventions to encourage appropriate STAT image ordering. METHODS/STUDY POPULATION: A fishbone diagram helped reveal possible causes of inappropriate STAT image ordering. Based on the fishbone diagram, a survey was created to assess CT image ordering habits amongst radiology and internal medicine residents and attending physicians. All CT Chest/Abd/Pelvis images ordered over a 3 month period of time (July-Oct 2017) was obtained. The dataset included whether the image was ordered Stat vs Routine, time of image order, physician name and location, and reason for the imaging study.The STAT images were evaluated based on the explanation provided in the CT image order. Currently 2 radiology residents, 2 internal medicine residents, and 2 internal medicine hospitalists are evaluating all STAT CT images to determine appropriateness and how long they are willing to wait for the image to result in a read. RESULTS/ANTICIPATED RESULTS: Analysis of all CT Chest/Abd/Pelvis imaging orders revealed that 51% (1710/3345) of them were ordered as STAT. The preliminary analysis of 227 STAT images showed that 6% were inappropriate. We anticipate results of our survey to show differences in how long a STAT vs Routine image orders should take amongst Radiology and Internal Medicine clinicians. We also anticipate our survey to show differences in factors that warrant STAT imaging amongst the different medical fields. We anticipate that the clinician manual evaluation of all STAT CT image will reveal a large percentage of imaging orders to be inappropriate. All STAT imaging that were flagged as inappropriate will be characterized by the department who ordered the image and the reason provided for the imaging to assess for common themes. DISCUSSION/SIGNIFICANCE OF IMPACT: STAT images are the new routine with more images ordered STAT than Routine. Inappropriate STAT imaging results in truly urgent patients not getting the medical care they need. Many images ordered stat could potentially be switched to routine. By evaluating why clinicians are ordering STAT CT image inappropriately, we will be able to develop targeted interventions to decrease inappropriate STAT CT imaging.

